# Editorial: Decoding Brain Function Through Genetics

**DOI:** 10.3389/fgene.2022.874350

**Published:** 2022-04-11

**Authors:** Kazuya Toriumi, Guang-Zhong Wang, Stefano Berto, Noriyoshi Usui

**Affiliations:** ^1^ Schizophrenia Research Project, Department of Psychiatry and Behavioral Sciences, Tokyo Metropolitan Institute of Medical Science, Tokyo, Japan; ^2^ Shanghai Institute of Nutrition and Health, University of Chinese Academy of Sciences, Chinese Academy of Sciences, Shanghai, China; ^3^ Department of Neuroscience, Medical University of South Carolina, Charleston, SC, United States; ^4^ Department of Neuroscience and Cell Biology, Graduate School of Medicine, Osaka University, Osaka, Japan; ^5^ United Graduate School of Child Development, Osaka University, Suita, Japan; ^6^ Global Center for Medical Engineering and Informatics, Osaka University, Osaka, Japan; ^7^ Addiction Research Unit, Osaka Psychiatric Research Center, Osaka Psychiatric Medical Center, Osaka, Japan

**Keywords:** neurogenomics, epigenetics, transcriptome, brain function, neurodevelopmental disorders (NDDs), psychiatric disorders, single nucleotide polymorphism (SNP), polygenic risk score (PRS)

Recently, several attempts have been made to explore the mechanisms underlying brain function using neurogenomics (Kang et al., 2011; Snyder et al., 2020; Yuste et al., 2020; Jourdon et al., 2021). Neurogenomics offers comprehensive perspectives on the profound impact of genomic alterations on brain function and facilitates an understanding of the complex interactions between genetics and the surrounding environment. It also aids in understanding the pathogenesis of brain disorders such as neurodevelopmental disorders (NDDs) and psychiatric disorders (Bogdan et al., 2013; Smoller et al., 2019; Pattabiraman et al., 2020; Lee et al., 2021). Our research presents a broad range of insights into the neurogenomic techniques used to decode brain physiology and pathophysiology (NDDs and psychiatric disorders). Specifically, this research topic included functional assessments of genetic polymorphisms and epigenetic studies using patient genome data or polygenic risk scores (PRSs) and comprehensive transcriptome studies using brain disease-related mouse models.

Firstly, studies discussing the discovery of single nucleotide polymorphisms (SNPs) or PRSs associated with brain diseases and their pharmacological effects were introduced. Wei et al. demonstrated that the rs187406 C>T polymorphism in the endothelin receptor type A gene (*EDNRA*) increased the susceptibility to large artery atherosclerotic stroke (Wei et al.). Iino et al. identified schizophrenia-associated SNPs in the aldo-keto reductase family 1 member A1 gene (*AKR1A1*). Although one of the SNPs located on the first nucleotide of the exon is a silent mutation, it results in exon skipping, thereby decreasing *AKR1A1* expression and functional activity (Iino et al.). Kasai et al. showed that short tandem repeats (STRs) in the cannabinoid receptor 1 gene (*CNR1*) were associated with analgesic requirements post-orthognathic cosmetic surgery (Kasai et al.). Blasi et al. showed that the rs363043 C>T SNPs of the synaptosomal-associated protein 25 (*SNAP25*) gene in children with borderline intellectual disability had reduced perceptual reasoning index and intelligence quotient (IQ) scores (Blasi et al.). In a cohort study of the general Japanese population, Takahashi et al. reported that the adult body mass index (BMI)-PRSs are associated with working memory in children (Takahashi et al.). This association was found to be related to reduced cortical thickness in the left inferior parietal lobe and left superior serratus gyrus. The abovementioned studies provide novel insights into the genetic factors associated with brain dysfunction, such as SNPs, STRs, and PRSs.

Secondly, two reports that evaluated DNA methylation as an epigenetic alteration were introduced. Fujisawa et al. reported a relationship between genome-wide methylation differences and brain structure in the development of attention-deficit hyperactivity disorder (ADHD) (Fujisawa et al.). Their study identified 61 methylation sites in monozygotic twins discordant for ADHD and showed increased methylation rates in the sortilin-related VPS10 domain-containing receptor 2 gene (*SORCS2*). Furthermore, the methylation rate in *SORCS2* was associated with a decrease in the gray matter volume in the precentral and posterior orbital gyri, which are both involved in language processing and emotional control. Nishitani et al. defined the aging rate based on DNA methylation frequency and analyzed the relationship between reproductive efforts (parity status, number of deliveries, motherhood period, and cumulative motherhood period) and the aging rate (Nishitani et al.). They showed that increased reproductive effort was associated with slower aging in mothers with four or fewer children. Nishitani et al. reported that increased left precuneus gray matter volume mediated the relationship between parity status and aging deceleration, suggesting that mothers may benefit from aging deceleration due to structural changes in the precuneus. Thus, therapeutic and preventive strategies targeting alterations can be pursued by identifying epigenetic alterations related to disease onset. Epigenomics is important in understanding the onset of psychiatric disorders that are strongly influenced by environmental factors.

Finally, three reports on animal models of brain disorders using genetic analysis were introduced. Usui et al. reported that postnatal mice exposed to stress in their early life demonstrated social deficits, anxiety-like behavior, and abnormalities in cell architecture, such as a decrease in the number of neurons in the prefrontal cortex (Usui et al.). RNA sequencing identified 15 genes related to transcriptional regulation, stress, and synaptic signaling in the stressed group, indicating that these changes caused behavioral deficits. Lim et al. found increased neuropilin-1 (*NRP1*) expression in the brain and blood of model mice with Alzheimer’s disease (AD) and the postmortem brain tissue of patients with AD (Lim et al.). Lim et al. suggested that the increased *NRP1* expression in the elderly may be responsible for the increased severity of severe acute respiratory syndrome coronavirus 2 (SARS-CoV-2) infection, suggesting that this gene is involved in the infectivity of the SARS-CoV-2 virus. Xie et al. reported that heterozygous mice with a serotonin transporter binding protein, N-ethylmaleimide-sensitive factor (Nsf), showed autism spectrum disorder-like behaviors and impairments of synaptic plasticity and glutamate-serotonin neurotransmission in the hippocampus (Xie et al.). Next-generation sequencing is a powerful tool that can provide comprehensive transcriptome analysis that will assist researchers in exploring the molecular mechanisms underlying brain diseases. Future studies combined with those from the latest single-cell transcriptome and epigenomics (Armand et al., 2021; Diez and Sepulcre, 2021) will provide more in-depth knowledge on the molecular and cellular mechanisms of brain diseases and dysfunction.

In conclusion, decoding the brain function through genetic analysis is in its nascent stages. Further technological developments will deepen our understanding of the role of genomics in brain function while simultaneously adding to its complexity. Currently, there is a gap between genomic observations and actual brain function. Therefore, future studies should take up the challenge of exploring the undiscovered brain functions. However, the diversity of the papers on this research topic leads us to believe that genetics has great potential to enhance our understanding of brain function.
